# Diverse coordinate frames on sensorimotor areas in visuomotor transformation

**DOI:** 10.1038/s41598-017-14579-3

**Published:** 2017-11-02

**Authors:** Yusuke Fujiwara, Jongho Lee, Takahiro Ishikawa, Shinji Kakei, Jun Izawa

**Affiliations:** 1 Brain Information Communication Research Laboratory Group, Advanced Telecommunications Research Institute International, Kyoto, 619-0288 Japan; 2 Motor Disorders Project, Tokyo Metropolitan Institute of Medical Science, Tokyo, 156-8506 Japan; 3 Faculty of Engineering, Information and Systems, University of Tsukuba, Ibaraki, 305-8573 Japan

## Abstract

The visuomotor transformation during a goal-directed movement may involve a coordinate transformation from visual ‘extrinsic’ to muscle-like ‘intrinsic’ coordinate frames, which might be processed via a multilayer network architecture composed of neural basis functions. This theory suggests that the postural change during a goal-directed movement task alters activity patterns of the neurons in the intermediate layer of the visuomotor transformation that recieves both visual and proprioceptive inputs, and thus influence the multi-voxel pattern of the blood oxygenation level dependent signal. Using a recently developed multi-voxel pattern decoding method, we found extrinsic, intrinsic and intermediate coordinate frames along the visuomotor cortical pathways during a visuomotor control task. The presented results support the hypothesis that, in human, the extrinsic coordinate frame was transformed to the muscle-like frame over the dorsal pathway from the posterior parietal cortex and the dorsal premotor cortex to the primary motor cortex.

## Introduction

Performing a visuomotor transformation is essential to allow the brain to produce motor commands to generate a goal-directed movement toward a visual target. The neural mechanism underlying this function has often been formalized by the coordinate transformation between the extrinsic coordinate frame that represents the visual target location in the external of the body and the intrinsic coordinate frame that represents the motor commands based on the muscle kinematics^1^. Ample neurophysiological data suggest that this transformation is mediated by gain fields represented in the posterior parietal cortex (PPC) and in the dorsal premotor cortex (PMd), in which the neural activities are representing the target as well as the movement directions, are modulated by the postural information^2,3^. A model prediction of a neural network model of visuomotor transformation^4–6^ is compatible with measurements of the gain field of the PPC neurons, whose tuning functions are modulated by the hand location and the eye location^7,8^. Extrapolating from these computational models, the neurons of the input layer represents information either on the intrinsic ‘proprioceptive’ coordinate frames or the extrinsic ‘visual’ coordinate frames and those of the output layer represents it on the intrinsic ‘muscle’ coordinate frames while the neurons of intermediate layer does not rely on any explicit coordinate frame but, as a result of integration of two input layers, it appears to depend on an intermediate coordinate frames. However, it is as yet unclear how exactly these diverse coordinate frames are represented in multiple cortical regions in the human brain. While this question is fundamental to understanding the neural mechanism of the coordinate transformation, it remains unsolved.

Investigating the coordinate frames of the regional activity of each cortical area aligned on the dorsal stream is essential to understand the neural mechanism of visuomotor transformation. The recently established multivariate analysis of the BOLD signals allows us to assay the selectivity of the neural population. In particular, the tuning function over the motor movement directions that appears in the multi-voxel activity pattern has been found in the primary motor cortex(M1), PMd, the primary sensory area (S1), and PPC^9,10^. These results support the theoretical prediction that a collection of the neural activities inside a certain voxel following the tuning functions of the population code form a tuning function of the BOLD signals and, thus, the directional tuning of the stimulus-related-activity can be read out from the multi-voxel pattern of the BOLD signals^11^. Based on this idea, previous papers have decoded motor commands as well as target representations from the multi-voxel activity pattern of the visuomotor areas using fMRI decoding^12–15^. During a center-out reaching task, this fMRI decoding technique clearly dissociated the coordinate frame of M1 from PMd and PPC, exhibiting directional tuning functions over the movement and target directions^16,17^. These results suggest that fMRI decoding enables us to capture the gain fields formed by population coding. While the previous papers reported the posture dependencies of the decoding accuracy^18^, it has not been examined yet which coordinate frame such as the extrinsic or the intrinsic frame is represented in each cortical area.

In this study, we established a decoding model to estimate coordinate frames from voxel patterns and applied it to human fMRI data during the experimental paradigm developed by Kakei *et al*.^19^, where their monkey subject conducted an isometric center-out wrist movement toward eight target directions across two forearm postures. To identify the coordinate frame of the wrist muscles of human, we conducted an intramuscular electromyography (iEMG) experiment separately from the fMRI experiment. We then measured fMRI data from human participants who underwent this task. Changing the posture for each target led the participants to produce two muscle activation patterns for each single target. This alters the multi-voxel activity pattern of the cortical area that encodes information in the intrinsic (muscle-like) coordinate frame, but preserves it in the area that encodes information in the extrinsic coordinate frame.

## Results

**Figure 1:**
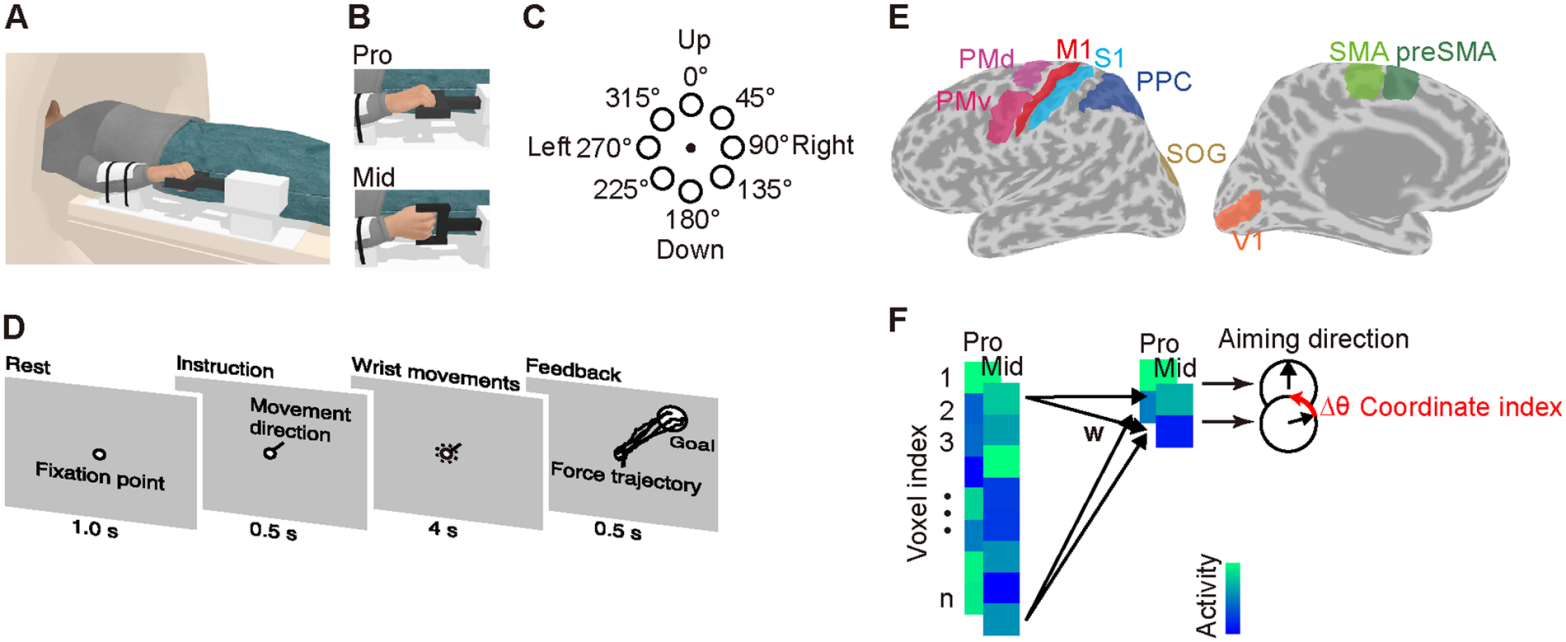
Overview of the decoding analysis for the visuomotor control task. (**A**) An MR-compatible force sensor mounted on the bed of the MRI scanner recorded the hand force produced by the wrist muscles. An arm cuff connected to the bed prevented participants from using their shoulder and the elbow muscles. (**B**) The task was conducted under two postures: pronated (Pro) and midway (Mid), which was between the pronated and the supinated postures. (**C**) The coordinate frame of the task space: the extension under the Pro postural condition (i.e., up for the participant) was defined as 0°. (**D**) The visual stimulus on the screen positioned in front of the participant’s face inside the scanner. Visual feedback of the aiming accuracy was provided by displaying force trajectories and the force target position. (**E**) Regions of interest. (**F**) A schematic of the model-based examination of coordinate frames. The 90° supination/pronation of the wrist altered the multi-voxel activity pattern of a region of interest (ROI) as well as the decoded pattern, which led to the rotation of the decoded aiming direction. Estimation of the rotation of the decoded direction Δ*θ* indexes the coordinate frame of the embedded information in the multi-voxel activity pattern of the ROI. A rotation of 0° in the predicted direction indicates the extrinsic coordinate frame, and a rotation of 90° indicates the joint coordinate frame.

We recorded fMRI data while participants underwent an isometric wrist force control task where they were asked to manipulate a cursor that represented their hand force (i.e., a force cursor). The force cursor was displayed on a screen mounted inside the MRI scanner, so that the cursor could reach inside the visual target while the hand force was measured by the in-house MRI-compatible force sensor (Fig. 1A–D). Note that the arm cuff prevented participants from using their shoulder and elbow muscles, and thus forced them to control only their wrist muscles. A task block with a 6-s duration began with the presentation of one of the eight target directions (0, 45, …, 315°), which was followed by four repetitive step-like cursor movements toward the visually instructed aiming direction (Fig. 1C and D). This isometric force control task was conducted in two forearm postural conditions: the pronated (Pro) posture and the midway (Mid) posture, which was supinated approximately 90° from the Pro posture and thus was in between the pronated posture and the supinated posture (Fig. 1B and C). The generated reach was directed to the presented target unbiasedly (Fig. 1D): The mean biases to the instructed directions across participants were −2.2° ± 7.0° and −6.4° ± 4.8° in Pro and Mid postural conditions, respectively.

**Figure 2:**
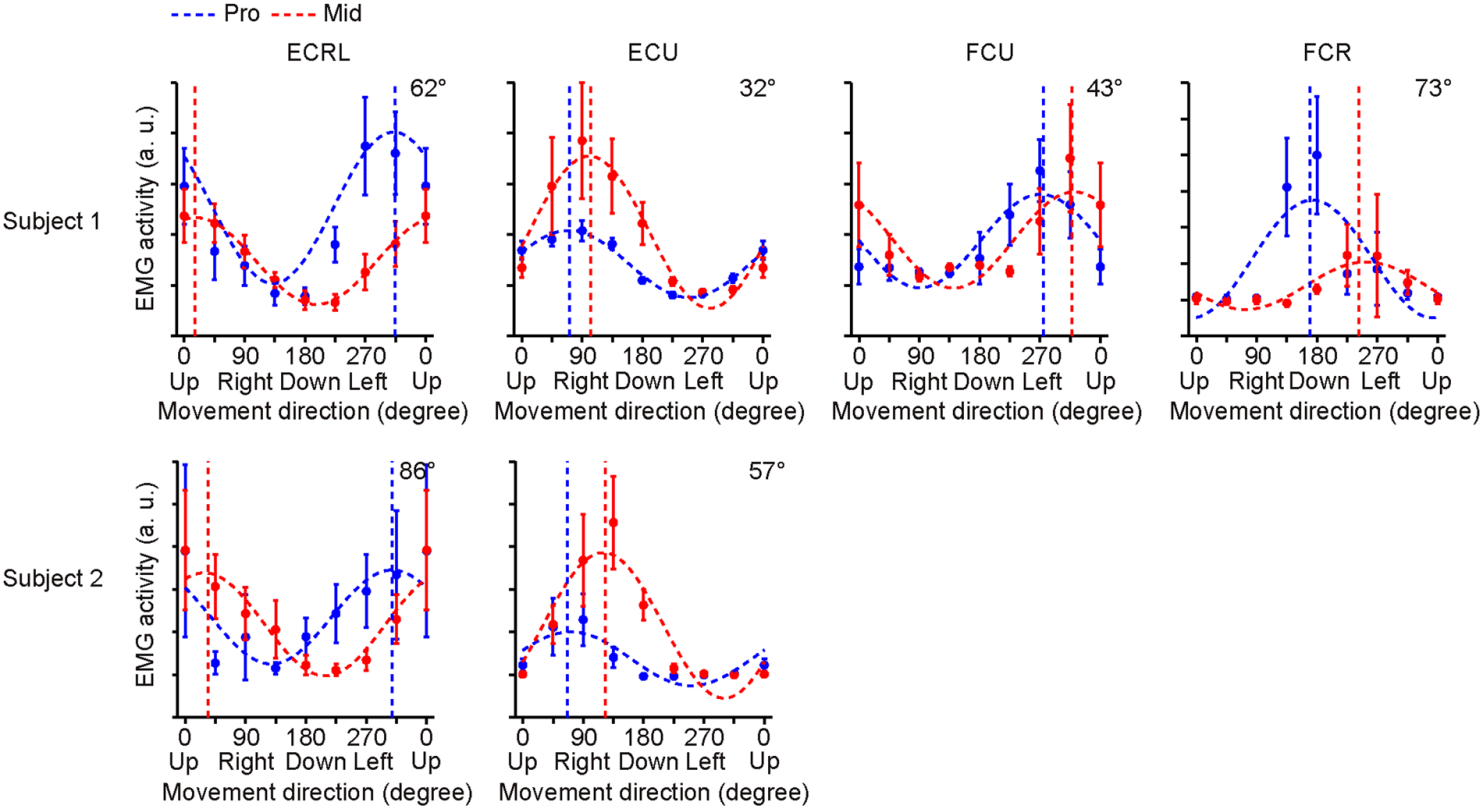
Cosine-tuning of muscle activities in different postures. Activities of the four forearm muscles (ECRL, ECU, FCU and FEC) observed from participant #1 and the two forearm muscles (ECRL and ECU) in participant #2 were measured during isometric wrist movements. The average activities relative to the rest period were calculated for each direction and each posture (blue, Pro; red, Mid). The filled circles with the error bars indicate the average and the 95% confidence intervals over the 10 trials. A cosine function was fitted to estimate the preferred direction of the muscle. The vertical dotted lines are the estimated preferred directions. The numbers in the upper right corner indicate shifts of the preferred directions from Pro to Mid postural conditions.

In advance of this fMRI experiment, we identified the coordinate frame of the wrist muscles of human participants, which was estimated as the rotation angle of the preferred direction (PD) of the wrist muscles caused by a posture change. Intramuscular electromyography (iEMG) was measured from two participants while they performed a similar isometric wrist control task as that in the scanner. Note that the participants in this study do not overlap with those in the fMRI experiment. The tuning function of the muscle activity was estimated from the four representative muscles (ECRL: extensor carpi radialis longus; ECU: extensor carpi ulnaris; FCR: flexor carpi radialis; FCU: flexor carpi ulnaris) in the two postural conditions (Pro and Mid). We found that the PD of the muscle was rotated 58.8° ± 18.3° clockwise when the posture changed from Pro to Mid (Fig. 2 and Methods section in detail). This estimated rotation angle is consistent with the previously reported intermediate coordinate frame between the extrinsic-like (0°) and the joint-like (90°) coordinate frames in the monkey. Note that, in this paper, we refer to the coordinate frame attached to the external of the body which is uninfluenced by the postual change of the wrist as the extrinsic coordinate frame, and it attached to the wrist which significantly rotates as the wrist rotation as the intrinsic coordinate frame. We also categorize the intrinsic coordinate frame by two: one that rotates exactly the same extent as the joint rotation as the joint-like coordinate frame and the other that rotates intermediately between the extrinsic and the joint as the muscle or proprioceptive coordinate frame.

### Postural sensitivity to the directional tuning property of the decoder

**Figure 3:**
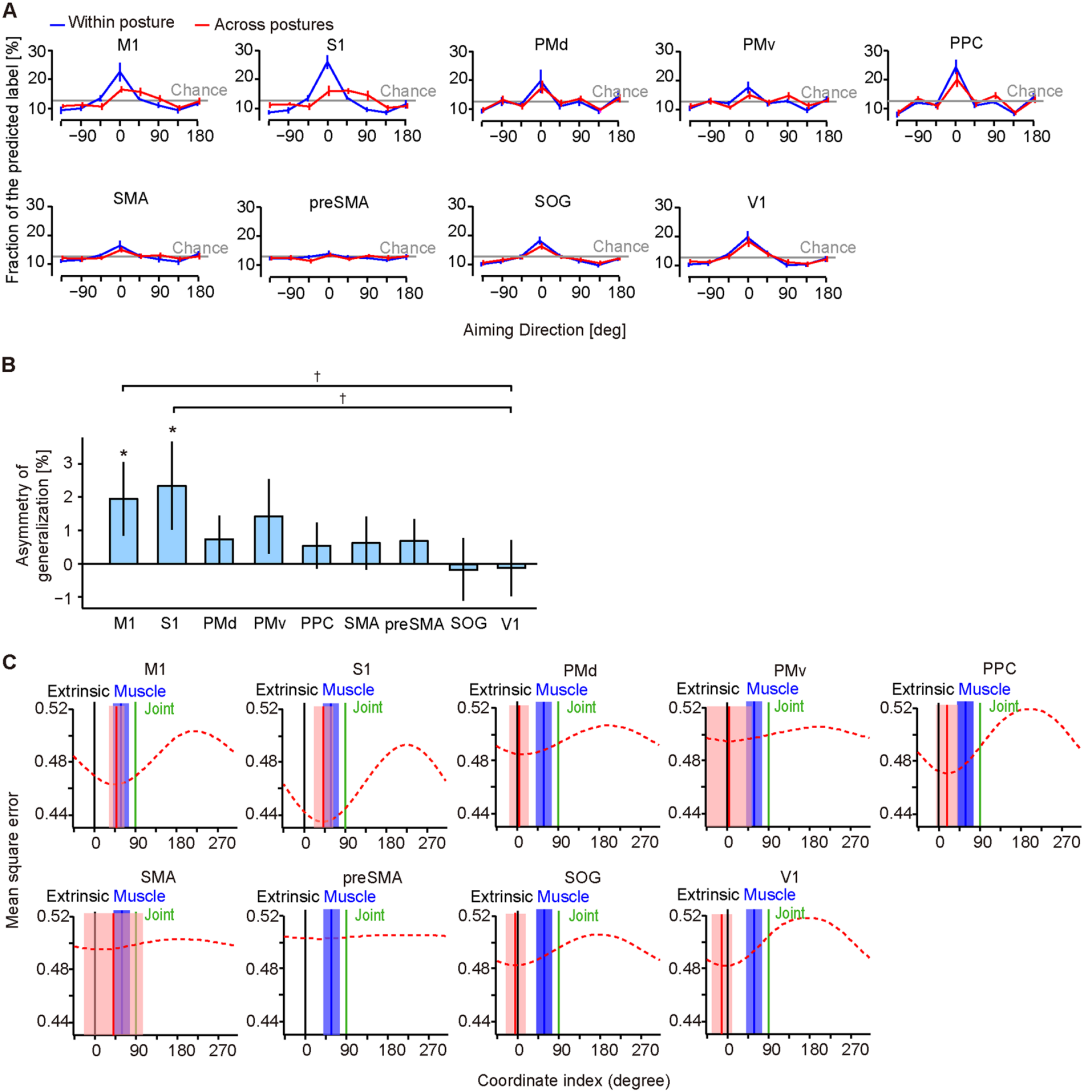
The tuning properties of the decoder over the aiming directions and the coordinate frames embedded in each ROI. (**A**) The across-participant mean with 95% CI of the fraction of the predicted direction of the decoder on each ROI. All predicted directions were aligned with respect to the tested true direction centered at 0°. Of the within-posture generalization (Blue), the trained and the tested multi-voxel activities were taken under the same postural condition. Of the across-posture generalization (Red), the trained and the tested multi-voxel activities were taken under different postural conditions. The gray solid line indicates the chance level of the prediction. (**B**) The bar graph of the difference of the asymmetry index between the within- and the across- postural conditions (across-participant mean ± 95% CI), where the asymmetry index was evaluated as the difference between the averaged prediction errors over the positive angles (45°, 90° and 135°) minus those over the negative angles (−45°, −90° and −135°). Asterisks indicate a significant effect of the postural condition on the asymmetry index (paired t-test, p < 0.01, Bonferroni corrected). Daggers indicate a significant difference from V1 (paired t-test, p < 0.01, Bonferroni corrected). (**C**) The prediction accuracy of the decoder with the across-posture rotation model (Equation 1) is plotted over the coordinate rotation angle Δ*θ* (red dashed line), which provides the best estimate of the rotation angle ${\rm{\Delta }}\hat{\theta }$ (red vertical solid line) that indexes the coordinate frame of the reference frame as the rotation angle from the extrinsic coordinate frame (0°, black). The rotation angle of the joint coordinate frame is 90° (green) and that of the muscle-like coordinate frame estimated from the iEMG data is 58.8° (blue). Every plotted line is the across-participant average, with the shaded area showing 95% CI.

We then analyzed postural selectivity of the multi-voxel patterns in anatomically defined ROIs (see Methods): M1, S1, PMd, The left ventral premotor cortex (PMv), PPC, the supplementary motor area (SMA), the superior occipital gyrus (SOG), the pre-supplementary motor area (preSMA), and the primary visual cortex (V1); see Table 1 for Talairach coordinate. First, we trained and tested on a decoder built in each ROI by the leave-one-run-out cross-validation to compute the fraction of predicted labels under the same postural condition (e.g., Pro). We then plotted them, aligned with respect to the instructed aiming label (i.e., the true label) of the tested data. With this analysis, we aimed to illustrate the within-posture generalization property of the built decoder over all aiming directions. As expected, in all ROIs except preSMA, the profile of the decoding generalization profile showed a single peak (PD, preferred direction) at the trained aiming direction with a moderate tuning curve (Fig. 3A, blue), which reminds us of the tuning properties of single neurons in these cortical areas^19–21^.

**Table 1:** Across-participant average Talairach coordinates of ROI.

ROI name	x	y	z
M1	−35	−16	40
S1	−40	−18	45
PMd	−23	−2	50
PMv	−49	6	22
SMA	−7	−12	41
preSMA	−10	14	37
PPC	−23	−54	48
SOG	−13	−89	14
V1	−12	−84	−9

Second, we trained the decoder on the data taken under one of the two postural conditions (e.g., Pro) and then tested it on the data taken under the other posture (e.g., Mid), and vice versa, to illustrate the across-posture generalization property. If a certain ROI represents information in the extrinsic coordinate frame, a posture change should not affect the decoding property. In fact, the overlap of the V1 tuning function between the within-posture and across-posture generalizations indicates that the aiming direction might be represented in the extrinsic coordinate frame, which is congruent with previous literature on the retinotopic organization of V1^22^. However, if a certain ROI represents information in the intrinsic coordinate frame, the postural change might alter the multi-voxel activity pattern of this ROI and then bias the generalization profile. For M1 and S1, the fraction of the trained label (0°) exhibited a remarkable decrease, while those for the positive 45° and 90° showed an increase as if the PD of the tuning function shifted towards the right (Fig. 3A, red). This apparent rotation of the PD appeared in M1 and S1 suggests that the coordinate frame representing aiming direction is attached to the forearm and rotates as the forearm rotates, indicating that these coordinate frames are more intrinsic-like than extrinsic-like.

We then evaluated this postural sensitivity to the generalization property by taking the difference between the average of the decoding accuracy over the positive angles (45°, 90° and 135°) and the negative angles (−45°, –90° and –135°) and compared this asymmetry index between the within-posture and the across-posture conditions (Fig. 3B). Because the postural conditions significantly altered the asymmetry indexes of M1 and S1 (t-test, p < 0.01, Bonferroni corrected) and these were significantly different from that of V1 (paired t-test, p < 0.01, Bonferroni corrected), these two areas might adopt dissociable coordinate frames from that of V1’s extrinsic coordinate frame. In contrast, because the sensitivity of PMd, PMv, PPC, SMA and preSMA, and SOG were not significantly different from that of V1, the extrinsic-like coordinate frames might be employed in these six areas.

### Decoder-based identification of coordinate frame

Extrapolating from these results, we hypothesized that, if the observed PD’s rotation is mainly caused by the postural influence on the encoded cortical information, we might be able to build a decoder that can predict the presented visual aiming direction from the fMRI data taken across two postural conditions by simply rotating the predicted aiming direction (Fig. 1F). Here, we call this decoder for two postural conditions a ‘common decoder’. If the coordinate frame of a certain ROI is extrinsic, the estimated rotation angle of the coordinate frame should be zero, whereas if it is attached to the forearm, it should be the same angle as that of the posture rotation (i.e., 90°). Thus, the estimated rotation angle quantifies how far the coordinate frame of the ROI is from the extrinsic coordinate frame, and thus enables us to index the coordinate frame embedded in this ROI.

We employ a linear decoder that transforms the N-dimensional vector of the voxel to the two-dimensional aiming direction. The common decoder **w** (2 × *N* vector; *N* is the number of voxels) on the multi-voxel activity patterns of both the Pro **r** ^Pro^ (*N* × 1 vector) and the Mid **r** ^Mid^ (*N* × 1 vector) conditions predicts the vectorial representation of the aiming direction **v** (2 × 1 vector) defined in the extrinsic frame when the direction predicted from the multi-voxel activity pattern of the Mid condition is rotated by *Δθ*,

1 $${\bf{v}}={\bf{w}}{{\bf{r}}}^{{\rm{Pro}}}={\rm{R}}({\rm{\Delta }}\theta ){\bf{w}}{{\bf{r}}}^{{\rm{Mid}}}$$

where $${\rm{R}}({\rm{\Delta }}\theta )=[\begin{array}{cc}cos{\rm{\Delta }}\theta  & -sin{\rm{\Delta }}\theta \\ sin{\rm{\Delta }}\theta  & cos{\rm{\Delta }}\theta \end{array}]$$ is the rotation matrix (Fig. 1F).

We estimated the decoder **w** with varying Δ*θ* from –45° to 315° to find the best estimate of the coordinate index ${\rm{\Delta }}\hat{\theta }$. This best estimate of the rotation angle ${\rm{\Delta }}\hat{\theta }$ indexes the coordinate frame of each ROI. The extrinsic coordinate frame is indexed with ${\rm{\Delta }}\hat{\theta }={0}^{^\circ }$, the joint coordinate frame is indexed with ${\rm{\Delta }}\hat{\theta }={90}^{^\circ }$, and, the muscle coordinate which laid in between these two^19^ is indexed, in this paper, with ${\rm{\Delta }}\hat{\theta }={58.8}^{^\circ }$ according to the iEMG data.

The dotted red line of Fig. 3C illustrates the across-participant average of the mean square error of the predicted aiming vector over the tested coordinate index Δ*θ* that provides the best estimate of this index ${\rm{\Delta }}\hat{\theta }$ at the minimum of this error function. The best estimate of the coordinate index of M1 is 50.0° ± 16.9° (mean angle ± 95% Confidence interval [CI]), which is significantly different from both the extrinsic coordinate frame and the joint coordinate frame (one sample test for circular variable, p < 0.001) and appears to be overlapped with the muscle-like coordinate frame. The index of S1 (42.0° ± 21.0°) also overlaps with the muscle-like coordinate frame, and those of PMd (4.6° ± 21.1°, p = 0.60), PMv (4.6° ± 49.5°, p = 0.85), and V1 (19.0° ± 22.3°, p = 0.30) are not different from the extrinsic coordinate frame. Then, the index of PPC (19.0° ± 22.3°) lies in between the extrinsic and the muscle-like. The error functions of SMA and preSMA are almost flat, and the estimated coordinate indices have a wide confidence interval.

## Discussion

By conducting the decoding analysis, we identified both the coordinate frame used in each cortical area. We found that both S1 and M1 represent the aiming vector in the muscle-like coordinate frame, PMd represents it in the extrinsic coordinate frame, and PPC represents in in the t intermediate between the extrinsic and the muscle-like coordinate frames.

To develop this multi-voxel pattern analysis to estimate coordinate frames, we hypothesized that a collection of the neural tuning functions in a certain cortical area that codes the visual target/movement direction should form an intrinsic multi-voxel activity pattern. We then built a decoder of the multi-voxel activity pattern to predict the visual target/movement vector over the two postures and directly estimated the angular difference between these two predicted vectors in the different postures. This estimated angular difference represents a coordinate index that enabled us to evaluate the significance of the coordinate rotation. Importantly, the estimated coordinate indices are consistent with the monkey neurophysiology literature: the muscle-like coordinates observed in neurons in M1^19^ and S1^21^, as well as the extrinsic coordinates seen in PMd^23,24^. This consistency supports our working hypothesis that the coordinate frame embedded in the cell activities characterizes the posture dependent alteration of the multi-voxel activity pattern.

In PPC, we found the intermediate coordinate frame. This is also consistent with the previous results, where the monkey PPC contains cells that represent an intermediate coordinate frame between the hand-centered coordinate frame and the eye-centered coordinate frame^25,26^. In theory, the intermediate coordinate may contribute to converting signal representations ascending from different sensory modalities, and thus the intermediate coordinate frame might indicate a middle layer’s representation of the multi-sensory integration process^4–6,27,28^. Hence, the intermediate reference frame appeared in the voxel patterns of PPC, might be a signature of the PPC’s contribution for the intermediate-stage representation of the visuomotor transformation via multi-sensory integration.

We should note that the wide confidence interval appeared in SMA and pre-SMP does not necessary mean that these two areas do not code any movement-related information or have any specific coordinate frames. The low decoding accuracies and the wide confidence interval might depend on the signal to noise ratio of voxels in ROIs.

Taken together with cortico-cortical connections from PMd and PPC to M1 found in monkey neuroanatomical studies^29,30^ as well as diffusion MRI tractography study in humans^31^, the projection from PMd and PPC to M1 might contribute to the coordinate transformation from the extrinsic or the intermediate frames to the muscle-like frames. These connections might play an important role in the feedback control^32–34^ based on online sensory inputs and the feedforward control^35^ unaffected by sensation. Further analyses would shed light on this question.

## Methods

### Participants

Twelve right-handed volunteers (2 female, mean age 25 ± 4.5 years old) without any history of the neurological disorder and with normal or corrected-to-normal vision participated in the fMRI experiment. An additional two right-handed healthy volunteers (male 33 and 38 years old) participated in the intramuscular EMG experiment. All participants gave written informed consent before participating in this study. The experimental paradigm was approved by the Institutional Review Board of Advanced Telecommunications Research Institute International and was conducted according to the Declaration of Helsinki.

### fMRI experiment

Inside the scanner, the participants grasped the handle of the in-house made MR compatible force sensor with their right hand, where their shoulder and elbow movement were restricted by a cuff that was mounted on the scanner bed. They were asked to push the handle toward the aiming direction, which was instructed by a short line starting from the fixation point along the requested aiming direction with a size of 1° of visual angle. The task was started with the presentation of an indicator for 0.5 s, and then the fixation point flickered four times over 4 s, i.e., once every 1 s. After the aiming direction was presented, the participant pushed the handle four times, following the end of the flicker of the fixation point. Subsequently, the visual feedback of the produced force trajectories was presented for 0.5 s, where 1° in visual angle corresponded to 1 N of measured wrist force. Meanwhile, the visual feedback of the required wrist force was presented with a circle 1° in radius located along the aiming direction, 5° from the center (i.e., 5 ± 1 N). After this feedback signal, a 1 s rest period was inserted, so that the duration of each block was 6 s. In each scan run, composed of 32 blocks, the target aiming direction was randomly selected every trial from eight directions, so that each direction appeared four times in total. A 20 s rest period was inserted at the beginning of each run to measure the baseline activity, and another 6 s rest period was added at the end of each run to allow for the time constant of the hemodynamic response. The participants experienced either the Pro or Mid postural condition for the first consecutive 7–9 runs and then experienced the other posture for the next consecutive 7–9 runs, to give 14–18 runs total. The order of the experienced postural conditions was counterbalanced across the participants.

### MRI Data Acquisition and Preprocessing

MRI data were obtained using a 3.0-T Scanner (Siemens MAGNETOM Verio) with a 32-channel head coil located at the ATR Brain Activity Imaging Center. fMRI signals were scanned with a gradient-echo echo-planar imaging (EPI sequence; TR = 2000 ms; TE = 26 ms; flip angle = 80°; field of view = 192 × 192 mm; voxel size = 3 × 3 × 3 mm; slice gap = 0 mm; number of slices = 33), covering the entire cerebral cortex. T1-weighted high-resolution (MP-RAGE sequence; field of view = 256 × 256 mm; voxel size = 1 × 1 × 1 mm; slice gap = 0 mm) structural images of the whole brain were acquired to define anatomical ROIs.

The fMRI data were subjected to 3D motion correction and were then aligned with the T1 structural image using SPM8. The fMRI signals of each voxel were shifted by 4 s to compensate for hemodynamic delays. The amplitudes of the signals were normalized by the mean amplitude of the first 20-s rest period in each run to minimize the baseline difference across runs. The temporal pattern of the fMRI signals underwent linear trend removal within each run. To define anatomical ROIs for each participant, the T1 structural image was transformed to Talairach coordinates and also converted to a flattened cortical surface using the Freesurfer software^36^. The voxels in the ROIs were transformed back into the original coordinates of the EPI images. The EPI voxels of each volume in each ROI was z-transformed to minimize the magnitude of activation between the different postural conditions.

### MRI Compatible Force Sensor

The two-degree-of-freedom (2DOF) MRI-compatible force sensor was constructed of a non-magnetic elastic material (acetal polyoxymethylene), an optical strain gauge composed of a digital fiber sensor (FS-N10; Keyence corp.) and a limited-reflective fiber unit (FU-38; Keyence corp.). The compatibility was confirmed by a measurement with a phantom brain in advance of the fMRI experiment. The wrist forces were recorded at 500 Hz. Movement directions were determined using the force trajectories derived from more than 50% of the maximum force of each movement.

### Intramuscular electromyogram (iEMG) Experiment

We performed an EMG experiment outside of the MRI scanner to evaluate the human muscle coordinate frame. The experiment was conducted at the Tokyo Metropolitan Institute of Medical Science and the protocol was approved by the Ethical Committee of the Tokyo Metropolitan Institute of Medical Science. We adopted the intramuscular EMG to measure each wrist muscle precisely, avoiding the contamination of the other muscle activities. Pairs of single-stranded stainless steel wires were inserted transcutaneously into four wrist muscles (ECRL, ECU, FCU and FCR) for one participant, and two extensor wrist muscles (ECR and ECU) for the other participant. We identified each muscle by observing the twitch of the muscle from an electrical stimulation (10 pulses at 200 Hz, 60–600 µA). EMG signals were recorded at 1000 Hz and band-pass filtered from 3–150 Hz. EMG data were normalized by data obtained during the rest period before movement onset (1 s). The participants lay on a bed in the supine position and performed isometric wrist movements in eight directions with a force of 5 N in the Pro and Mid postural conditions. The participants maintained a force of 5 N for 5 s in each direction and conducted this movement 10 times in each direction with each posture.

The preferred direction (PD) of each muscle was calculated by averaging the EMG of 1 s after the muscle force became stable at 5 N. The average amplitudes were fitted by a cosine function^20^,

2 $$f(\theta )=k\,cos(\theta \,-\,{\rm{\mu }})+b,$$

and the peak direction *μ* was estimated as the PD of the muscle. A circular mean and 95% CI across the 6 muscles’ PDs as a muscle-like coordinate frame were estimated using the MATLAB Toolbox for Circular Statistics^37^.

### ROI Selection

Eight anatomical ROIs (left hemisphere) were defined using anatomical landmarks^38,39^ on the flattened cortical surface with Freesurfer^36^. M1 voxels were selected from the anterior wall of the central sulcus and the posterior bank of the precentral gyrus. S1 voxels were selected from the posterior wall of the central gyrus and the anterior bank of the postcentral gyrus. The premotor cortex (PMd and PMv) was defined on the precentral sulcus and the anterior bank of the precentral gyrus, and PMd and PMv were separated at z = 50 in Talairach coordinates^40–42^. SMA and pre-SMA were defined on the medial wall extended from y = −20 to y = 20 ^43^ and the border of SMA and pre-SMA was set at y = 0 ^44,45^. PPC was defined as the superior parietal lobule where the most anterior border is the postcentral sulcus, the most posterior border is the parieto-occipital sulcus and the interior border is the intraparietal sulcus (IPS). SOG voxels were selected from the superior occipital gyrus with reference to a recent fMRI study^46^. V1 voxels were selected from the calcarine sulcus^22^ as a control region.

### Decoding of Target Label

To analyze the alternation in multi-voxel activity pattern during the task which was provoked by the changes in aiming direction and posture, we used the linear SVM and examined the alternation in the fraction of predicted labels regarding aiming directions. The multi-voxel activity patterns of each volume (TR = 2) of each ROI was used. The fraction of predicted labels with the confidence interval was computed with leave-one-run-out cross-validation.

### Estimation of Coordinate Frame

To identify the coordinate index of each ROI, the aiming direction as a continuous value was decoded from the multi-voxel activity pattern as the two-dimensional vector using linear regression. The estimation of the common decoder $\hat{{\bf{w}}}$ was given by the sparse linear regression^47^ with changing Δ*θ* with 5° step between 40° to 315°. Among all pairs of $\hat{{\bf{w}}}$ and Δ*θ*, we chose optimal parameters $\hat{{\bf{w}}}$ and ${\rm{\Delta }}\hat{\theta }$ with which the prediction error of the aiming vector was minimum. The performance of the common decoder $\hat{{\bf{w}}}$ with the best estimate ${\rm{\Delta }}\hat{\theta }$ was evaluated using the nested cross validation.
